# Sense of Parenting Efficacy, Perceived Family Interactions, and Parenting Stress Among Mothers of Children With Autistic Spectrum Disorders

**DOI:** 10.3389/fpsyg.2022.878158

**Published:** 2022-04-28

**Authors:** Yirong Chen, Tianyi Cheng, Fangyan Lv

**Affiliations:** ^1^School of Educational Science, Quan Zhou Normal University, Quanzhou, China; ^2^School of Psychology, Nanjing Normal University, Nanjing, China; ^3^Department of Psychology, Guangdong Provincial Key Laboratory of Social Cognitive Neuroscience and Mental Health, Sun Yat-sen University, Guangzhou, China; ^4^School of Marxism, Sun Yat-sen University, Guangzhou, China

**Keywords:** mothers of children with autistic spectrum disorder, sense of parenting efficacy, perceived family interactions, parenting stress, moderation effect

## Abstract

This study examined the relationship between maternal sense of parenting efficacy and parental stress in children with autism and the moderating effect of family interaction. A total of 263 mothers of children with autism were investigated with the Parenting Ability Scale, Family Interaction Scale (FIS), and Parental Stress Scale. The results showed that (1) maternal sense of parenting efficacy significantly predicted parental stress in children with autism; and (2) family interaction significantly moderated the relationship between maternal sense of parenting efficacy and parental stress in children with autism, that is, when family interaction was lower than −1.54 standard deviation (SD), the sense of parenting efficacy did not significantly predict parental stress. When family interaction was higher than −1.54 SD, parenting efficacy had a significant negative predictive effect on parenting stress.

## Introduction

Autism spectrum disorder (ASD) is a complex neurodevelopmental disorder characterized by varying degrees of language development disorder, interpersonal communication disorder, repetitive behavioral pattern, and narrow interest (McKinnon et al., 2019). Autism begins in infancy and lasts for a lifetime. Parents often face persistent stressful situations and experience high parental pressure (Hsiao, 2018). In China, the prevalence of ASD is increasing to more than 1% of the total population, and the estimated population of children (aged 0–14 years) with autism is more than 2 million (Clark et al., 2019). Parenting stress refers to the stress parents feel in the process of fulfilling their role, affected by their personality traits, parent–child interaction, children's traits, and family situation and accompanied by negative psychological feelings of anxiety, frustration, and self-blame (Abidin, 1990). Mothers, as the primary caregiver of children with ASD, usually face a variety of problems, such as a heavy financial burden, discrimination, stigma, and parenting stress (Abidin, 1992). Parenting stress not only affects marital relationship, intergroup relationship, physical and mental health, and family life quality, but also has a higher risk for poor treatment outcomes (Chen et al., 2015; Ban and Sun, 2017; Schlebusch et al., 2017; Zhao et al., 2017; Sim et al., 2018).

The sense of parenting efficacy is a factor identified as relevant to parent distress and child therapy outcomes (Liu, 2019). Many empirical studies on parents of children with autism in China have found that the sense of parenting efficacy has significant predictive effects on parental stress and subjective wellbeing (Lei et al., 2010; Wang et al., 2020; Zhu et al., 2020a). The sense of parenting efficacy is defined as an individual's subjective perception of parenting ability and confidence when playing the role of parents (Peng et al., 2012). The sense of parenting efficacy is considered to be the core factor affecting the educational effectiveness of children with autism, which can significantly predict the parenting ability of children with autism and promote active participation in parenting (Solish and Perry, 2008). According to the Bandura's self-efficacy theory (Bandura, 1978), the sense of parenting efficacy affects individuals' emotional states and coping styles. Mothers with a high-level sense of parenting efficacy can choose more appropriate type of therapy for their children. On the contrary, mothers with a low-level sense of parenting efficacy are more likely to experience negative emotions, such as tension and anxiety. The sense of parenting efficacy, as an individual's internal psychological resource, can relieve stress and promote positive emotional experience, and also enable parents to experience significantly different parental stress in the process of raising children with autism (Iadarola et al., 2018; Güler and Letin, 2019). Mothers with a high-level sense of parenting efficacy tend to have a higher willingness and motivation to intervene, which can promote the active participation of family members in parenting and contribute to the children therapy (Benson et al., 2008; Yao and Liu, 2018). Based on this, this study hypothesized that the parenting sense of competence of mothers is an important factor affecting parenting stress.

The ecosystem theory divides the environment of individual life into different levels. Among them, the family is the microsystem with the largest physical and mental development of family members of children with autism, and the interaction between family members affects individual psychology and behavior (Loveland, 2001; Bronfenbrenner and Morris, 2006; Xiong and Sun, 2014; Buchanan et al., 2017; Martinez-Torres et al., 2021). Therefore, individual psychology and behavior may be affected by situational factors (family interaction) in family ecosystem. Family interaction is the degree to which family members care for and support each other in their daily life (Fang et al., 2004). Parents' participation in upbringing and good family interaction between parents and children can build a good environment for children with autism, reflect the family's acceptance of children and the openness of family members, and help to promote the formation of a good educational force. In addition, the family provides certain environmental conditions for the healthy development of family members. The family is an important support system for the parents of children with autism, which provides certain environmental conditions for the healthy development of family members in physiology, psychology, and sociality (Skinner and Steinhauer, 2000; Fang et al., 2004; Prendeville and Kinsella, 2019; Degli Espinosa et al., 2020). Good family interaction and family support help to rebuild self-worth and play a crucial role in promoting the confidence of individual parenting. At the same time, the language input of parents in family interaction is a kind of language stimulation for children with autism and the application of language communication strategies in family interaction can promote the development of children's communication and language (Ye et al., 2020). Previous studies have also confirmed that parental participation in parenting and parent–child interaction is considered to be important factors to alleviate parental pressure with a low-level sense of parenting efficacy (Shumow and Lomax, 2002; Jones and Prinz, 2005; Weiss et al., 2016; Zhou et al., 2019; Benedetto et al., 2021; Feng et al., 2021; Kurzrok et al., 2021). Parenting efficacy is an important factor for the effectiveness of autism parenting training programs (Russell and Ingersoll, 2020). It has been supported that interventions for families with children with ASD should focus on enhancing parental self-efficacy (Feng et al., 2021). Therefore, this study hypothesized that parental involvement and parent–child interaction are considered to be important factors in alleviating parental stress with a low parenting sense of competence.

In conclusion, this study was based on family ecosystem theory (Eppler, 2019), interaction theory, and self-efficacy theory (Bandura, 1978). Involving the mother of children with autism as the research object, this study aimed to explore the moderating effect of family interaction on the relationship between parenting sense of competence and parental stress in mother, so as to provide a reference for the development of family support services for children with autism. Considering previous studies, it is found that age and family structure are significantly correlated with parental pressure of parents of children with ASD (Liu, 2019; Hu and Guan, 2020). Therefore, these variables are controlled.

## Materials and Methods

### Participants

We randomly contacted 18 special education schools serving children with autism in Mainland China. Notably, 15 of 18 schools expressed interest to participate in the research. Random sampling was used, and the samples were representative. Participating in this study required the parents to have a child diagnosed with ASD by a certified doctor according to the DSM-IV-TR criteria (American Psychiatric Association, 2000). Although the ASD diagnosis is based on parent self-report, a clinician's report was required for the child to get enrolled in the special education schools. Excluding invalid questionnaires, the final sample included 263 children with ASD, and their mothers were recruited in the study. There were 225 boys (85.6%) and 38 girls (14.4%). The mean [standard deviation (SD)] age of the children was 5.64 (2.63) years, ranging from 2 to 15 years; most of them aged 3–7 years (77.1%). Among 263 mothers, the mean (SD) age was 35.56 (4.42) years, ranging from 24 to 48 years; most mothers aged 30–40 years (82.5%). Of note, 256 (97.3%) mothers were married and only 7 (2.7%) mothers were divorced. Mothers with employment accounted for 41.1%, and 35.4% of them had a university degree education level. In addition, 70.3% of them reported that annual family income was US$8,000 to US$16,000, and 78 (29.7%) mothers reported that annual family income was more than US$16,000. No incentive was provided to families to complete the survey.

### Measures

In this study, three instruments were used to explore the relationship among mother's sense of parenting efficacy, family interaction, and parenting stress, namely, the Parenting Sense of Competence Scale (PSOC), the Family Interaction Scale (FIS), and the Parenting Stress Inventory—Short Form (PSI-SF).

### Parenting Sense of Competence Scale

The PSOC compiled by Johnston and Mash (1989), and revised by Peng et al. (2012), contains 12 items from the two dimensions of sense of parenting efficacy and parenting ability satisfaction. Using a 5-level score (with 1 being completely inconsistent, 5 being completely consistent), the higher the scale score means, the more confident they are in their parenting ability. The model fit index has χ^2^ = 178.31, *df* = 46, Goodness of Fit Index (GFI) = 0.92, Normed Fit Index (NFI) = 0.90, Incremental Fit Index (IFI) = 0.93, Tucker-Lewis Index (TLI) = 0.90, Comparative Fit Index (CFI) = 0.92, and Root Mean square Residual (RMR) = 0.08. Cronbach's α value for the PSOC was 0.77, and the values for the satisfaction and efficacy subscales were 0.85 and 0.84, respectively.

### Family Interaction Scale

The FIS in the Family Life Quality Scale compiled by the Bridge Disability Center at the University of Kansas has 6 items in total, mainly measuring the interaction degree of family members of the disabled (e.g., “when faced with difficulties, my family will solve the problem together”) (Poston et al., 2006). Each item is answered on a 5-point scale ranging from 1 = strongly disagree to 5 = strongly agree. The model fit index is χ^2^ = 14.83, df = 5, GFI = 0.98, NFI = 0.99, Relative Fit Index (RFI) = 0.97, IFI = 0.99, TLI = 0.98, CFI = 0.99, RMR = 0.02, and Root Mean Square Error of Approximation (RMSEA) = 0.08. Cronbach's α value of this subscale in this study was 0.92.

### Parenting Stress Inventory—Short Form

The PSI-SF compiled by Abidin (1990) and revised by Ren (1995) contains 36 questions from three dimensions, namely, parental distress, dysfunctional parent–child interaction, and difficult children. Using a 5-level score (with 1 being completely inconsistent, 5 being completely consistent), the higher the scale score, the more stress the parents experience in parenting. The model fit index is χ^2^ = 890.566, *df* = 574, IFI = 0.90, TLI = 0.90, CFI = 0.90, and RMSEA = 0.06. Cronbach's α values for the three subscales were 0.92, 0.88, and 0.86, respectively.

### Procedure

After obtaining informed consent, the special education teachers issued the paper questionnaires to the mothers of children with autism, uniformly introduced the guidelines, and informed the test contents and requirements. The questionnaire is anonymous to ensure the authenticity and reliability of the survey. The test time was about 15 min, and all questionnaires were collected on the spot. This study was approved by the Institutional Review Board at the first author's affiliation.

### Statistical Analysis of Data

The data obtained in this study were statistically analyzed using SPSS version 24.0 and Amos version 24.0, and the statistical methods used were mainly descriptive statistics, namely, confirmatory factor analysis and model test of regulating action. The data were sorted and statistically analyzed using SPSS version 24.0, and Amos version 24.0 was used for confirmatory factor analysis.

## Results

### Common Method Bias

We used Harman's one-factor test to determine whether the data exist common method. In this test, we used the SPSS factor analysis routine to identify the first eigenvalue from the data matrix. The test results reveal that the first eigenvalue accounts for 30.91% of total variances, which does not equate to the majority of the total variance explained (threshold of 40%). Thus, according to Harman's one-factor test, common method bias is not likely to bias the results (Zhou and Long, 2004).

### Descriptive Statistics

Table 1 shows the mean, SD, and correlation coefficient of each variable. As predicted, Pearson correlations demonstrated that both parenting sense of competence and perceived family interactions were negatively correlated with parenting stress. Parenting sense of competence was positively correlated with perceived family interactions.

**Table 1:** Description statistics and zero-order correlation of the constructs.

	***M***	***SD***	**1**	**2**	**3**	**4**	**5**
1. Age	35.56	4.42	—				
2. Family structure	1.74	0.75	0.08	—			
3. Parenting sense of competence	2.89	0.54	0.15^*^	0.08	—		
4. Perceived family interactions	3.26	1.05	0.07	0.03	0.24^***^	—	
5. Parenting stress	2.99	0.68	0.03	−0.23^*^	−0.37^***^	−0.41^***^	—

* *p < 0.05*,

*** *p < 0.001*.

### Moderate Inspection

We used model 1 of the PROCESS program using SPSS version 24.0 (moderate model). All variables except age and family structure were standardized to analyze whether family interaction moderated the relationship between sense of parenting efficacy and parental stress. Table 2 shows that, after controlling for age and family structure, sense of parenting efficacy significantly predicted parental stress (β = −0.38, *p* < 0.001), family interaction significantly predicted parental stress (β = −0.40, *p* < 0.001), and the interaction between parenting efficacy and family interaction significantly predicted parental stress (β = −0.14, *p* < 0.001). Therefore, the relationship between sense of parenting efficacy and parental stress is moderated by family interaction, which proves the hypothesis.

**Table 2:** Hierarchical regression analysis among study variables.

**Outcome variable**	**Predictor Variable**	**β**	***SE***	**95% CI**	***R^**2**^***	***F***
Parenting stress	Age	0.02^*^	0.01	(0.01, 0.05)		
	Family structure	−0.26^***^	0.06	(−0.38, −0.14)		
	Parenting sense of competence	−0.38^***^	0.05	(−0.49, −0.27)		
	Perceived family interactions	−0.40^***^	0.05	(−0.48, −0.29)		
	Parenting sense of competence × perceived family interactions	−0.14^**^	0.04	(−0.23, −0.04)	0.47	45.87^***^

* *p < 0.05*,

** *p < 0.01*,

*** *p < 0.001*.

**Figure 1:**
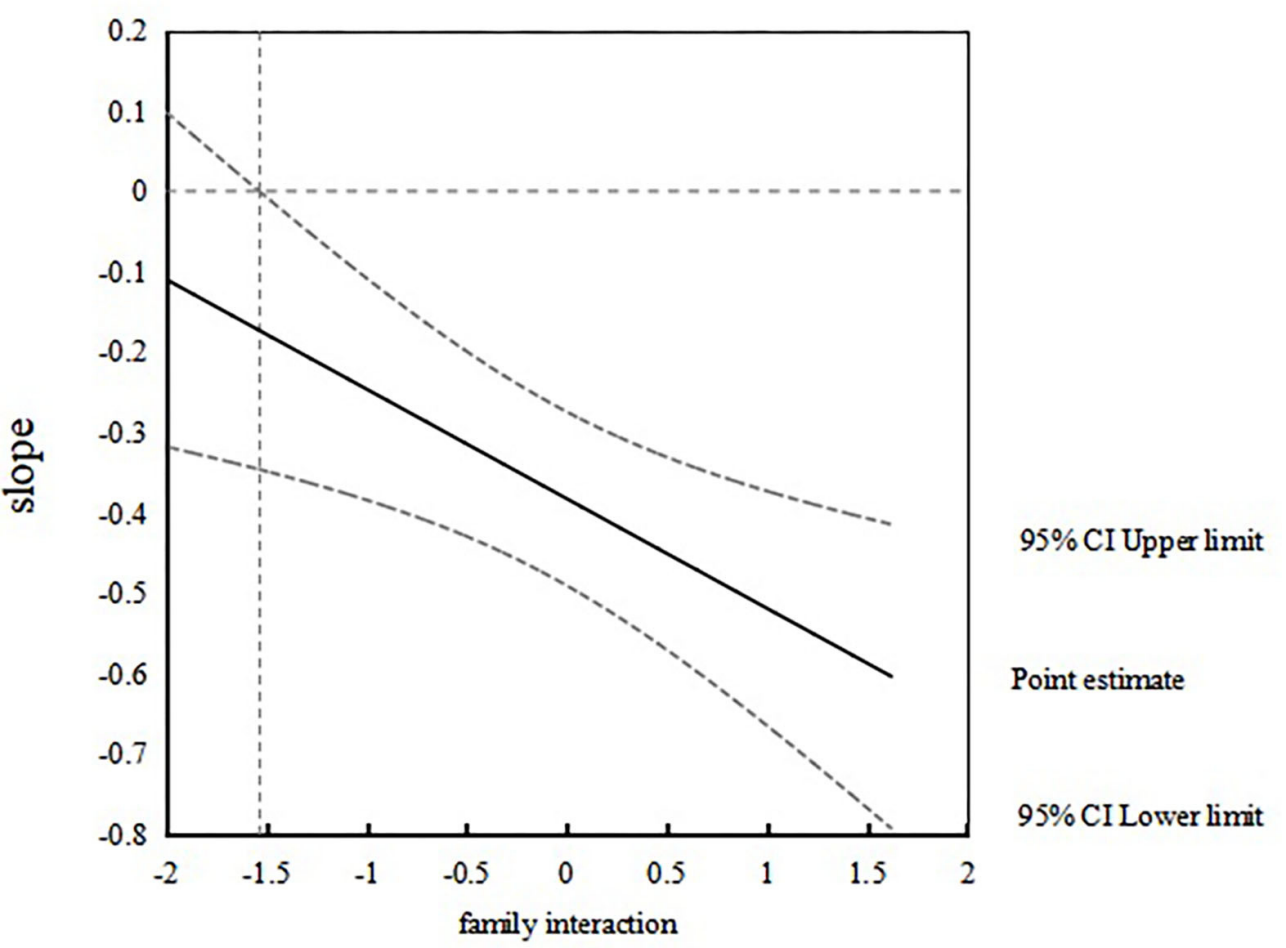
The effect of parenting sense of competence on relationship parenting stress.

The Johnson–Neyman method was used to investigate the relationship between sense of parenting efficacy and parental stress at different levels of family interaction (Fang et al., 2015), and a simple effect analysis chart was drawn. The results further demonstrated the moderating effect of family interaction. When the family interaction is lower than 1.54 SDs, the sense of parenting efficacy had no significant influence on parenting stress; when the family interaction is more than −1.54 SDs, the sense of parenting efficacy had significantly negative parenting stress. That is to say, with the increase in family interaction level, the effect of parenting self-efficacy on parental stress increases (refer to Figure 1), and the proportion of cases accounted for 90.49% of the sample size.

## Discussion

We investigated the relationship among mother's parenting sense of competence, family interaction, and parenting stress. We found that family interaction plays a moderating role in the relationship between maternal parenting efficacy and parental stress in mothers of children with autism.

This study found that the parenting sense of competence of mothers of children with autism significantly negatively predicted parental stress, which is consistent with the results of previous studies (Li et al., 2015; Kartini et al., 2018). This result is consistent with the view of self-efficacy. Parenting sense of competence is the subjective perception of mothers on the ability and confidence of raising children and affects individual emotional state and coping style. Mothers with a high parenting sense of competence can choose more appropriate type of therapy and respond to challenges more actively. On the contrary, mothers with low parenting efficacy are more likely to experience negative emotions, such as tension and anxiety. The sense of parenting efficacy, as an individual's internal psychological resource, can relieve stress and promote positive emotional experience, and can also enable parents to experience significantly different parental stress in the process of raising children with autism (Johnson et al., 2011). In ASD parenting training programs, parents can learn to get involved in the intervention in their natural context to promote the development and learning of their sons or daughters with ASD (Russell and Ingersoll, 2020). Thus, parents with greater parental efficacy are more involved in rehabilitation and treatment processes (Feng et al., 2021). Some studies have revealed improvement in the levels of parenting self-efficacy among parents who take part in psycho-educational interventions and parent training (Deb et al., 2020). A greater parenting sense of competence predicts better parent–child relationships and less parenting stress (Benedetto et al., 2021). Mothers with a high parenting sense of competence often have higher intervention willingness and motivation, can promote family members to actively participate in parenting, and play a vital role in children therapy (Benson et al., 2008; Yao and Liu, 2018). The results of this study also confirmed the viewpoint of resource conservation theory (Hobfoll, 2001; Kaniel and Siman-Tov, 2011; Hayward et al., 2020). According to the resource conservation theory, parental psychological resources can help people cope with stressors and improve the relationship between parents and their children in the context of interactions with them (Kaniel and Siman-Tov, 2011). Parents are more likely to experience perceived failure in a parenting task if their children are more difficult to parent due to problem behavior or poor emotional regulation (Teti and Gelfand, 1991). The sense of parenting efficacy was an individual's internal psychological resource (Iadarola et al., 2018). When individuals consume internal resources to meet their role needs, they will activate positive emotions, such as pleasure, happiness, and pride, so as to alleviate stress and promote positive emotional experience (Leary and Brown, 1995; Güler and Letin, 2019; Zhu et al., 2020b). Therefore, the sense of parenting efficacy of mothers of children with autism can significantly affect parental stress.

This study also found that family interaction regulates the relationship between sense of parenting efficacy and parental stress. The higher the level of family interaction, the greater the possible impact of sense of parenting efficacy on parental stress. Specifically, when the level of family interaction is lower than −1.54 SDs, the negative impact of sense of parenting efficacy on parental stress is not significant. When the level of family interaction is higher than −1.54 SDs, the sense of parenting efficacy has a significant negative predictive effect on parental stress. This implies that parenting efficacy has a greater impact on parental stress at higher levels of family interaction than that at lower levels of family interaction. The results confirm the view of family ecosystem theory (Robles-Bykbaev et al., 2017; Kassim et al., 2020; Zhao and Fu, 2020). An appropriate family ecosystem supports children with autism and their families, so as to improve their quality of life (Kassim et al., 2020), and supports the development of social communication skills in children with autism (Robles-Bykbaev et al., 2017). Family is an important promoter of education for children with autism. Good family interaction helps families play a vital role in seeking treatment, helping coordinate services between professionals, and jointly participating in education, which is an important driving force to promote children with autism to receive an education. Family interaction builds a good environment for children, reflects the family's acceptance of children with autism and the openness of family members, and is conducive to promote the formation of a good educational force. Family interaction and sense of parenting efficacy have a two-way impact. Specifically, parents' belief in improving children's educational effectiveness can effectively promote family interaction and educational participation. At the same time, the higher the degree of family interaction, while promoting educational participation, parents also gain parenting experience and promote the improvement of parental competence. Therefore, in the case of high family interaction, family members actively participate in parenting and interact with children with autism through interaction strategies or parents as intermediaries can have a positive impact on children's development (Shire et al., 2015; Shu et al., 2016; Xu et al., 2021), so as to further improve parents' parental sense of competence and effectively alleviate the parental pressure in the process of raising children by parents. Studies have supported parent-mediated intervention (Shu et al., 2016; Xu et al., 2021). In the case of low family interaction, the social and emotional feedback of children with autism to their caregivers is limited. On account of interacting with children with autism is a difficult thing, it is difficult for parents to maintain long-term interaction with children with autism. The lack of family interaction will lead to increased parenting pressure and reduced family cohesion.

This study found that family interaction plays a regulatory role in the relationship between parenting sense of competence and parental stress of children's mothers, which provided a new idea for further developing family support services for children with autism and alleviating parental stress of children's mothers. First, parenting efficacy has a significant negative predictive effect on parental stress of children with autism. This indicates that the mothers of children with autism with better parenting efficacy have less parental stress. Mothers are the primary caregivers of autistic children and the core of their social ecosystem. Authorities and social workers need to recognize the importance of mothers in the development of children with autism. In the relevant training for parents of autistic children, the main educational goal should be to cultivate mothers' sense of parenting efficacy, so as to reduce mothers' parenting pressure to the greatest extent. Second, family interaction is an important situational factor for family outcomes and family functioning. Family interaction affects individual psychology and behavior, which suggests that we should actively build family environmental factors for children with autism, stimulate family vitality by organizing family activities, promote benign interaction among family members, and enhance the acceptance and openness of family members to children with autism, promoting parenting sense of competence and alleviating parental pressure. Autism is not a static state in the human body, but a neurodevelopmental process, which should be understood from the interaction between human and environment.

Previous studies have found that the parenting efficacy of parents of children with autism can predict parental stress (Lei et al., 2010; Wang et al., 2020; Zhu et al., 2020a). This study has two contributions. One is that this study found that the parenting efficacy of mothers of children with autism negatively predicts parental stress, which verified previous studies. The other is that this study found that family interaction plays a regulatory role in the relationship between parenting sense of competence and parental stress of children's mothers. However, this study has the following limitations. (1) This study was only performed involving mothers, but some family functions cannot be fully borned by the mother and some problems of children with autism largely arise from a lack of emotional care from their father. In future research, we will compare the differences in father and mother participation. (2) The subject population may not be representative. There are two reasons. One is that the subjects selected for this study came from Fujian Province, which is an economically prosperous region in China. The other is that the sample size is not large enough. If the sample size is increased to more than 1,000, the obtained model data may be better. (3) This study was a cross-sectional study. The results indicated a possible mechanism and did not prove a causal relationship, which should need readers' attention.

Above all, further study should explore the role and impact of family interaction on children with autism and strive to optimize family interaction to the greatest extent to promote the development of children with autism. Future research may also pay more attention to the establishment of good family interaction and high-quality family interaction of children with autism and may deeply explore how the family interaction developed in a virtuous circle is formed and developed.

## Data Availability Statement

The raw data supporting the conclusions of this article will be made available by the authors, without undue reservation.

## Ethics Statement

The studies involving human participants were reviewed and approved by the Institutional Review Board at School of Educational Science, School of Special Education, Quan Zhou Normal University, Quanzhou, China. The patients/participants provided their written informed consent to participate in this study.

## Author Contributions

YC: designed research, collected data, and drafted manuscript. TC: designed research, analyzed data, and drafted manuscript. FL: designed research, analyzed data, and revised the manuscript. All authors certify that they have participated sufficiently in the work to take public responsibility for the content and approved the final version of the article for submission.

## Funding

This research was supported by the Youth Project of the Humanities and Social Science Fund of the Ministry of Education of the People's Republic of China (Grant No. 21YJC880010).

## Conflict of Interest

The authors declare that the research was conducted in the absence of any commercial or financial relationships that could be construed as a potential conflict of interest.

## Publisher's Note

All claims expressed in this article are solely those of the authors and do not necessarily represent those of their affiliated organizations, or those of the publisher, the editors and the reviewers. Any product that may be evaluated in this article, or claim that may be made by its manufacturer, is not guaranteed or endorsed by the publisher.
